# 洛拉替尼特殊不良反应管理中国专家共识

**DOI:** 10.3779/j.issn.1009-3419.2022.101.39

**Published:** 2022-08-20

**Authors:** 清 周, 舜 陆, 勇 李, 福军 贾, 冠军 李, 震 洪, 铀 卢, 云 范, 建英 周, 喆 刘, 娟 李, 一龙 吴, 中国胸部肿瘤研究协作组, 洛拉替尼特殊不良反应管理中国专家共识工作组

**Affiliations:** 1 510080 广州，广东省肺癌研究所，广东省人民医院，广东省医学科学院 Guangdong Lung Cancer Institute, Guangdong Provincial People' s Hospital, Guangdong Academy of Medical Sciences, Guangzhou 510080, China; 2 200030 上海，上海交通大学附属胸科医院肿瘤科 Department of Oncology, Shanghai Chest Hospital, Shanghai Jiao Tong University, Shanghai 200030, China; 3 200040 上海，复旦大学附属华山医院心内科 Department of Cardiology, Huashan Hospital, Fudan University, Shanghai 200040, China; 4 510120 广州，广东省精神卫生中心精神科 Department of Psychiatry, Guangdong Mental Health Center, Guangzhou 510120, China; 5 200030 上海，上海交通大学医学院附属精神卫生中心精神科 Department of Psychiatry, Shanghai Mental Health Center, Shanghai Jiao Tong University School of Medicine, Shanghai 200030, China; 6 200040 上海，复旦大学附属华山医院神经内科 Department of Neurology, Huashan Hospital, Fudan University, Shanghai 200040, China; 7 610041 成都，四川大学华西医院肿瘤科 Department of Oncology, West China Hospital of Sichuan University, Chengdu 610041, China; 8 310022 杭州，浙江省肿瘤医院肿瘤科 Department of Oncology, Zhejiang Cancer Hospital, Hangzhou 310022, China; 9 310003 杭州，浙江大学附属第一医院呼吸科 Department of Respiratory Diseases, The First Affiliated Hospital, College of Medicine, Zhejiang University, Hangzhou 310003, China; 10 101149 北京，首都医科大学附属北京市胸科医院肿瘤科 Department of Oncology, Beijing Chest Hospital, Capital Medical University, Beijing 101149, China; 11 610041 成都，四川省肿瘤医院肿瘤内科 Department of Oncology, Sichuan Cancer Hospital, Chengdu 610041, China

**Keywords:** 间变性淋巴瘤激酶, 肺肿瘤, 洛拉替尼, 不良反应, Anaplastic lymphoma kinase, Lung neoplasms, Lorlatinib, Adverse event

## Abstract

间变性淋巴瘤激酶（anaplastic lymphoma kinase, *ALK*）融合基因是非小细胞肺癌（non-small cell lung cancer, NSCLC）中第二常见的肿瘤驱动基因。作为新型的第三代ALK酪氨酸激酶抑制剂（tyrosine kinase inhibitor, TKI），洛拉替尼对多种*ALK*激酶域突变具有广谱且高效的临床活性，并具有强大的穿透血脑屏障效力。洛拉替尼的总体耐受性良好，其独特的不良反应或不良事件包括高脂血症与中枢神经系统反应等，多为轻至中度，通常经剂量调整和/或标准医疗干预即可管理。对于*ALK*阳性晚期NSCLC，开始洛拉替尼治疗前应充分评估患者基线特征与既往用药状况，预先告知患者可能经历的用药相关不良反应，并定期监测以实现药物临床获益的最大化。同时，多学科专家团队对于建立全面的诊断和治疗策略是至关重要的。

## Abstract (en)

Anaplastic lymphoma kinase (*ALK*) fusions represent the second most common oncogenic driver mutation in non-small cell lung cancer (NSCLC). As the new class of 3^rd^ generation of ALK tyrosine kinase inhibitor (TKI), lorlatinib has shown robust potency and brain-penetrant clinical activity against a wide spectrum of multiple resistance mutations within the ALK domain detected during crizotinib and 2^nd^ generation ALK TKI treatment. Lorlatinib is generally well-tolerated with unique adverse drug reaction/adverse event, including hyperlipidemia and central nervous system effects, which are mostly mild to moderate severity and manageable through dosage modifications and/or standard medical intervention. For advanced NSCLC with *ALK* positivity, patients should be evaluated for baseline characteristics and pre-existing medication, informed of the potential toxicities, and periodically monitored to balance benefits and risks. Moreover, a multidisciplinary group of experts is essential to establish a comprehensive diagnostic and therapeutic strategy.

## 1 前言

肺癌是全球发病率第二以及死亡第一的癌症^[1]^，也是我国发病率和死亡率最高的肿瘤。2022年估计我国新确诊病例870, 982例，死亡病例766, 898例^[2]^。肺癌中80%-85%为非小细胞肺癌（non-small cell lung cancer, NSCLC）^[3]^，69%的晚期NSCLC携带可靶向的基因突变，其中间变性淋巴瘤激酶（anaplastic lymphoma kinase, *ALK*）融合基因是第二常见的肿瘤驱动基因，占NSCLC患者的3%-7%^[4]^。

第一代和第二代ALK酪氨酸激酶抑制剂（tyrosine kinase inhibitor, TKI）开创了*ALK*阳性晚期NSCLC的治疗模式，但大多数患者在治疗后终将出现耐药，中枢神经系统（central nervous system, CNS）肿瘤进展是常见的进展部位^[5, 6]^。洛拉替尼是一种新型的第三代ALK TKI，采取大环分子结构设计，对克唑替尼和第二代ALK TKI治疗期间检出的多种ALK激酶域耐药突变具有广谱且高效的活性。洛拉替尼能够减少P-糖蛋白1介导的外排，因而具有强大的穿透血脑屏障效力^[7-9]^。

全球首个洛拉替尼III期随机对照试验CROWN研究^[10]^3年随访显示，在既往未接受过系统性治疗的晚期*ALK*阳性NSCLC患者中，与克唑替尼（*n*=147）相比，洛拉替尼（*n*=149）一线治疗使疾病进展或死亡风险下降73%[风险比（hazard ratio, HR）=0.27，95%置信区间（confidence interval, CI）：0.18-0.39]；3年无进展生存（progression-free survival, PFS）率：洛拉替尼64% *vs*克唑替尼19%，CNS进展风险下降92%（HR=0.08, 95%CI: 0.04-0.17）；基线伴可测量脑转移亚组中，CNS客观缓解率（objective response rate, ORR）：洛拉替尼83% *vs*克唑替尼23%。洛拉替尼在亚洲人群中也显示了相对于克唑替尼的疗效优势（ORR：76% *vs* 57%，12个月PFS率：72% *vs* 48%；CNS ORR：73% *vs* 25%）^[11]^。无论患者携带何种*EML4*-*ALK*变异类型或是否存在*ALK*激酶突变，洛拉替尼组在ORR、缓解持续时间和PFS方面均优于克唑替尼组^[12]^。

与其他ALK TKI类似，洛拉替尼的总体耐受性良好，但具有独特的不良反应（adverse drug reaction, ADR）或不良事件（adverse event, AE）特征。本共识将结合洛拉替尼在中国人群临床研究中的ADR或AE发生情况，根据循证依据和临床经验对其管理策略进行论述，旨在为临床医生提供参考。

## 2 洛拉替尼的安全性

洛拉替尼治疗期间，最常见的ADR或AE包括高脂血症、水肿、CNS反应、体重增加以及周围神经病变^[7, 13]^。与总人群相比，中国患者高脂血症、肝毒性以及高血糖发生率相对较高，而CNS和周围神经病变发生率较低（表 1）^[14]^。

**表 1:** 洛拉替尼治疗的晚期*ALK*阳性NSCLC患者中常见ADR或AE发生率（任何级别和3级-4级）

ADR或AE	总人群^[7, 13]^			亚洲和中国人群^[11, 14]^	
	任何级别^*^	3级-4级^*^		任何级别^*^	3级-4级^†^
高胆固醇血症	70%	16%		68%-93%	12%
高甘油三酯血症	64%	20%		68%-92%	28%
认知影响	21%	2%		2.8%-20%	0.9%
情绪影响	16%	2%		1.8%	NR
周围神经病变	34%	2%		17%-31%	0%
水肿	56%	4%		28%-44%	0.9%
体重增加	38%	17%		42%-54%	6.4%
ALT升高	17%	3%		43%	1.8%
AST升高	14%	2%		40%	2.8%
高血压	18%	10%		12%-22%	NR
高血糖	9%^‡^	3.2%^‡^		18%	2.8%
房室传导阻滞	1.9%^‡^	0.2%^‡^		NR	NR
ILD或非感染性肺炎	1.9%^‡^	0.6%^‡^		NR	NR
^*^治疗中出现的任何原因导致的AE。^†^治疗相关AE。^‡^数据基于对476例患者的汇总分析，包含CROWN研究的149例和NCT01970865研究的327例；其他总人群数据均源于CROWN研究结果。ADR：不良反应；AE：不良事件；ALK：间变性淋巴瘤激酶；ILD：间质性肺疾病；NR：未报告；NSCLC：非小细胞肺癌；ALT：天冬氨酸转移酶；AST：丙氨酸转移酶。					

全球人群中，洛拉替尼治疗期间严重AE发生率为34%，≥3级AE为72%，因AE而需暂停、减量或停药的患者比例分别为49%、21%和7%^[13]^；中国人群中上述结果分别为21%、61%、28%、12%和2.8% ^[14]^。

洛拉替尼相关ADR或AE多为轻至中度，通过剂量调整（调整方案见表 2）和/或适当的对症支持治疗可以在很大程度上得到缓解。肿瘤科医生应掌握常见和特殊ADR或AE的处理对策，通过合理监测、早期发现、及时干预等手段最终使ADR或AE后果最小化，患者获益最大化。鉴于ADR或AE涉及不同器官系统，多学科团队管理至关重要。

**表 2:** 洛拉替尼治疗的晚期ALK阳性NSCLC患者中针对常见ADR或AE的剂量调整方案^*^

ADR或AE	不同级别的剂量调整方案^[7]^			
	1级	2级	3级	4级
高胆固醇血症或高甘油三酯血症	-	-	-	应暂停用药，直至高胆固醇血症和/或高甘油三酯血症恢复至≤2级，之后以原剂量恢复治疗；若复发，则降低1个剂量水平再继续治疗
CNS反应	以当前剂量继续治疗。或暂停治疗直至缓解至基线水平，之后以原剂量恢复治疗或降低1个剂量水平继续治疗	暂停治疗直至缓解至≤1级，之后降低1个剂量水平继续治疗		永久性终止治疗
高血压	-	-	暂停治疗直至缓解至≤1级，之后以原剂量恢复治疗，或降低1个剂量水平继续治疗（若3级复发），或永久性终止治疗（若高血压经最佳医疗措施仍不能充分控制）	暂停治疗直至缓解至≤1级，之后降低1个剂量水平继续治疗，或永久性终止治疗（尤其对于4级复发）
高血糖	-	-	暂停治疗直至高血糖充分控制，之后降低1个剂量水平继续治疗，或永久性终止治疗（若高血糖经最佳医疗措施仍不能充分控制）	
房室传导阻滞^*^	一度：以当前剂量继续治疗； 二度：暂停治疗直至PR间期<200 ms，之后降低1个剂量水平继续治疗； 三度（完全）：暂停治疗直至安置起搏器或PR间期<200 ms，之后以原剂量恢复治疗（若安置起搏器）或降低1个剂量水平继续治疗（若未安置起搏器）； 三度（完全）复发：安置起搏器或永久性终止治疗			
ILD或非感染性肺炎	永久性终止治疗			
其他	以当前剂量或降低1个剂量水平继续治疗		暂停治疗直至症状缓解至≤2级或基线值，之后降低1个剂量水平继续治疗	
^*^洛拉替尼治疗NSCLC的推荐剂量方案为100 mg，每日1次整片吞服，伴或不伴食物，直至疾病进展或出现不可接受的毒性反应。允许降低1个或2个剂量水平，分别减至75 mg、50 mg，若需要进一步减量，则终止治疗^[7]^。^*^根据NCI CTCAE 5.0版（v5.0），一度、二度、三度（完全）房室传导阻滞分别分为1级-2级、1级-5级、2级-5级。一度无需剂量调整，二度和三度的剂量调整方案通用于全部级别。NCI：美国国立癌症研究所；CTCAE：不良事件通用术语标准。				

下文将分别讨论各个毒性反应的特征及其处理方式。全球总人群ADR或AE发生率的数据，绝大部分来自CROWN研究（*n*=149）；在该研究无数据的情况下，引用来自说明书的汇总分析结果（*n*=476，包含CROWN研究的149例和NCT01970865研究的327例），少数数据来自NCT01970865研究的阶段性报告（*n*=295）。

### 2.1 高脂血症

#### 2.1.1 发生率和临床特点

高脂血症是洛拉替尼治疗时最常见的ADR，中国患者发生率在90%以上，高于总人群（不超过70%）（表 1）。高胆固醇血症和高甘油三酯血症在总人群中至发生中位时间均为15 d^[7, 15]^，持续时间分别为451 d和427 d^[15]^。高脂血症较少导致剂量延迟（因高胆固醇血症或高甘油三酯血症需要暂停治疗的患者分别为4%和7%）、减量（1%和3%）（*n*=476）^[7]^或永久性终止治疗（总计1%）（*n*=149）^[13]^。而动脉粥样硬化性心血管疾病（atherosclerotic cardiovascular disease, ASCVD）的发病风险随着血总胆固醇或低密度脂蛋白胆固醇水平升高而增加；当血清甘油三酯 >500 mg/dL时，急性胰腺炎风险明显升高^[16]^。

#### 2.1.2 评估与分级

血总胆固醇或低密度脂蛋白胆固醇和甘油三酯水平作为血脂异常指标，在血脂分级基础上，判断患者发生ASCVD的风险。无论血清总胆固醇或低密度脂蛋白胆固醇水平如何，确诊的ASCVD患者均可直接列为极高危；当血清胆固醇≥288 mg/dL或低密度脂蛋白胆固醇≥190 mg/dL时，可直接列为高危。同时应考虑其他风险因素，如合并糖尿病、高血压、超重以及吸烟等，以全面评估或及时调整风险分层^[16]^。考虑到接受洛拉替尼治疗的*ALK*阳性晚期NSCLC患者的预期生存时间有望超过3年，应在制定抗肿瘤治疗策略前咨询心血管科医生，以针对特定人群进行高脂血症的长期危害评估。

#### 2.1.3 监测与管理

启动洛拉替尼治疗前根据是否伴有既存血脂异常及其程度进行分层，以确定患者能否开始治疗、是否需要开始降血脂干预或调整当前降脂药。在开始洛拉替尼治疗后1个月和2个月时，以及之后定期（每3个月一次）监测血清胆固醇和甘油三酯。若合并年龄（男性≥55岁；女性≥65岁）和/或达到ASCVD风险高危或极高危者，使用他汀类或他汀联合降脂药物治疗后，监测频率需增加至每1-3个月一次。

暴露于一定程度的高脂血症对患者并无即刻的危险，医生有时间窗在保持洛拉替尼当前治疗的情况下管理血脂水平^[9]^。然而，重度或以上甘油三酯升高（即 >500 mg/dL尤其 >1, 000 mg/dL）可能诱发急性胰腺炎，发生率约为14%，而触发急性胰腺炎的甘油三酯水平在既往经历过急性胰腺炎发作的易感患者中因人而异^[17]^。与高甘油三酯血症相关的胰腺炎可能是致命的，因此必须充分了解该风险以及患者潜在的遗传倾向^[17]^。对于危及生命的高脂血症，需暂停洛拉替尼，之后以原剂量恢复治疗；复发时，根据严重程度以原剂量或减量恢复治疗（图 1）^[7]^。

**图 1:**
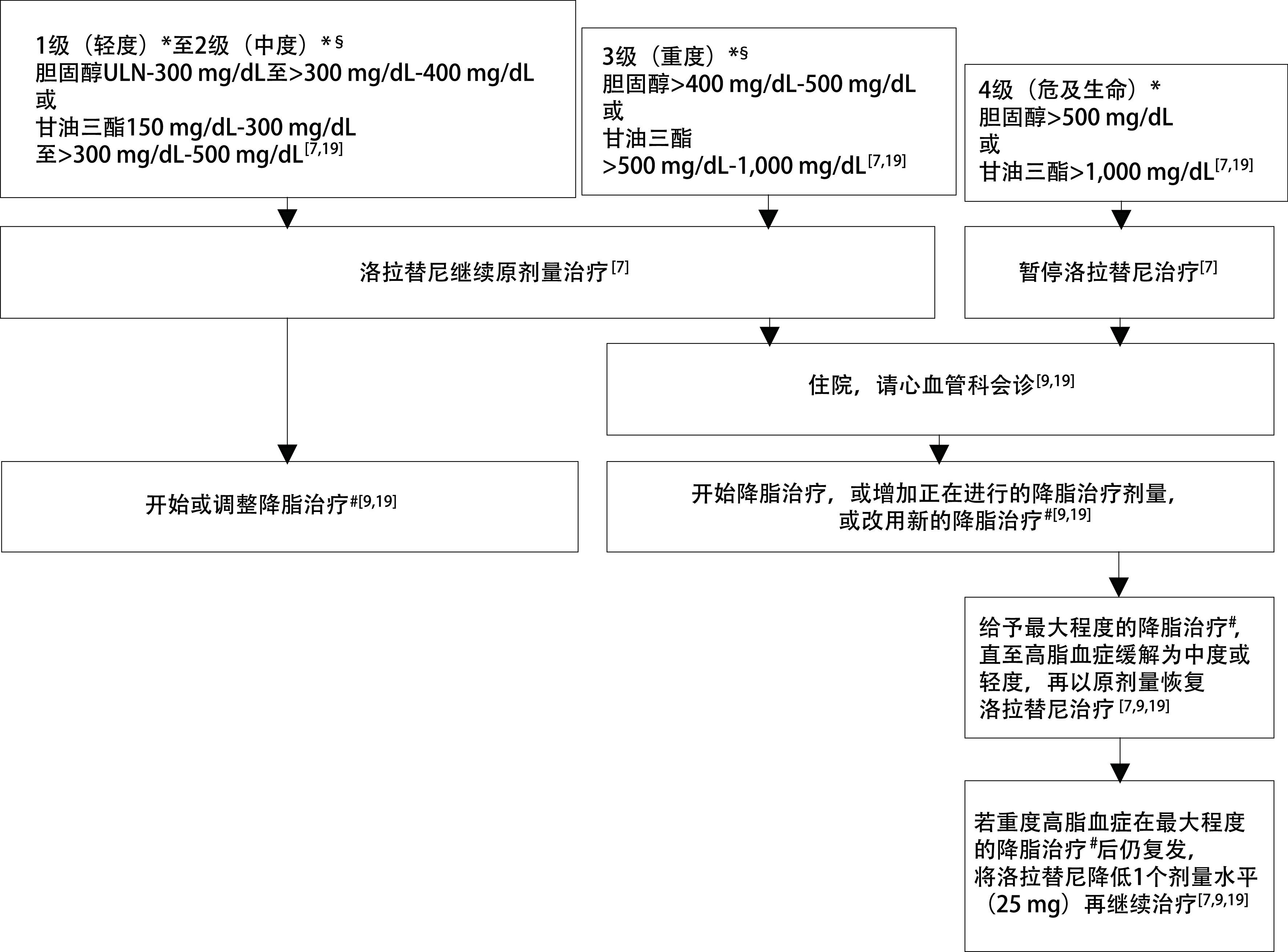
洛拉替尼相关高脂血症的管理流程。^*^应同时进行生活方式相关的靶点干预，包括戒烟、低饱和脂肪饮食、中等强度的体力活动以及减重等^[9, 18, 19]^；^§^甘油三酯>500 mg/dL意味着胰腺炎风险升高^[9, 18]^；^#^在他汀类基础上可联合依折麦布和/或非诺贝特，后两者分别在降低胆固醇和降低甘油三酯方面有优势^[9, 18, 19]^。ULN：正常值上限。

降低胆固醇或低密度脂蛋白胆固醇水平的治疗，应基于患者心血管风险分层设定个性化目标，具体心血管危险评估分层以及干预靶点和治疗目标请参照欧洲心脏病学会（European Society of Cardiology, ESC）和欧洲动脉粥样硬化学会（European Atherosclerosis Society, EAS）^[18]^以及中国血脂异常防治指南或中国血脂异常基层诊疗指南^[16]^。

无论高血脂源于基础疾病或继发于其他因素，还是洛拉替尼所致，降脂管理与洛拉替尼治疗是平行和并行的。高血脂和肿瘤治疗有各自独立的目标、疗程以及调整方法，他汀类降脂药物选择的核心原则为避免药物相互作用。首选瑞舒伐他汀、匹伐他汀或普伐他汀（表 3）；若需要在他汀类基础上增加治疗强度，可联合使用依折麦布或非诺贝特，次之可选择鱼油和烟酸；若他汀类联合非诺贝特治疗无效，可考虑依折麦布^[19]^（降低胆固醇或低密度脂蛋白胆固醇的联合降脂药物治疗首选他汀类+依折麦布）。吉非罗齐联合他汀类药物可增加肌病的风险，禁忌同时使用^[18, 19]^。非诺贝特、苯扎贝特或环丙贝特等其他贝特类与他汀类联合使用时，肌病的风险不会或几乎不会增加^[18]^。肌病为他汀类药物临床最相关的ADR，横纹肌溶解为最严重的肌肉损伤类型，发生率为1例-3例/100, 000患者-年，以重度肌肉疼痛、肌肉坏死和肌红蛋白尿为特征，可能导致急性肾功能衰竭和死亡^[18]^。他汀类药物治疗期间肌肉症状的管理流程见图 2^[18, 20]^。

**表 3:** 治疗洛拉替尼相关高脂血症的他汀类推荐剂量

药物	剂量^[21]^
匹伐他汀	2 mg，每日1次，口服
普伐他汀	40 mg，每日1次，口服
瑞舒伐他汀	5 mg-10 mg（中等强度），每日1次，口服; 20 mg（高强度），每日1次，口服

**图 2:**
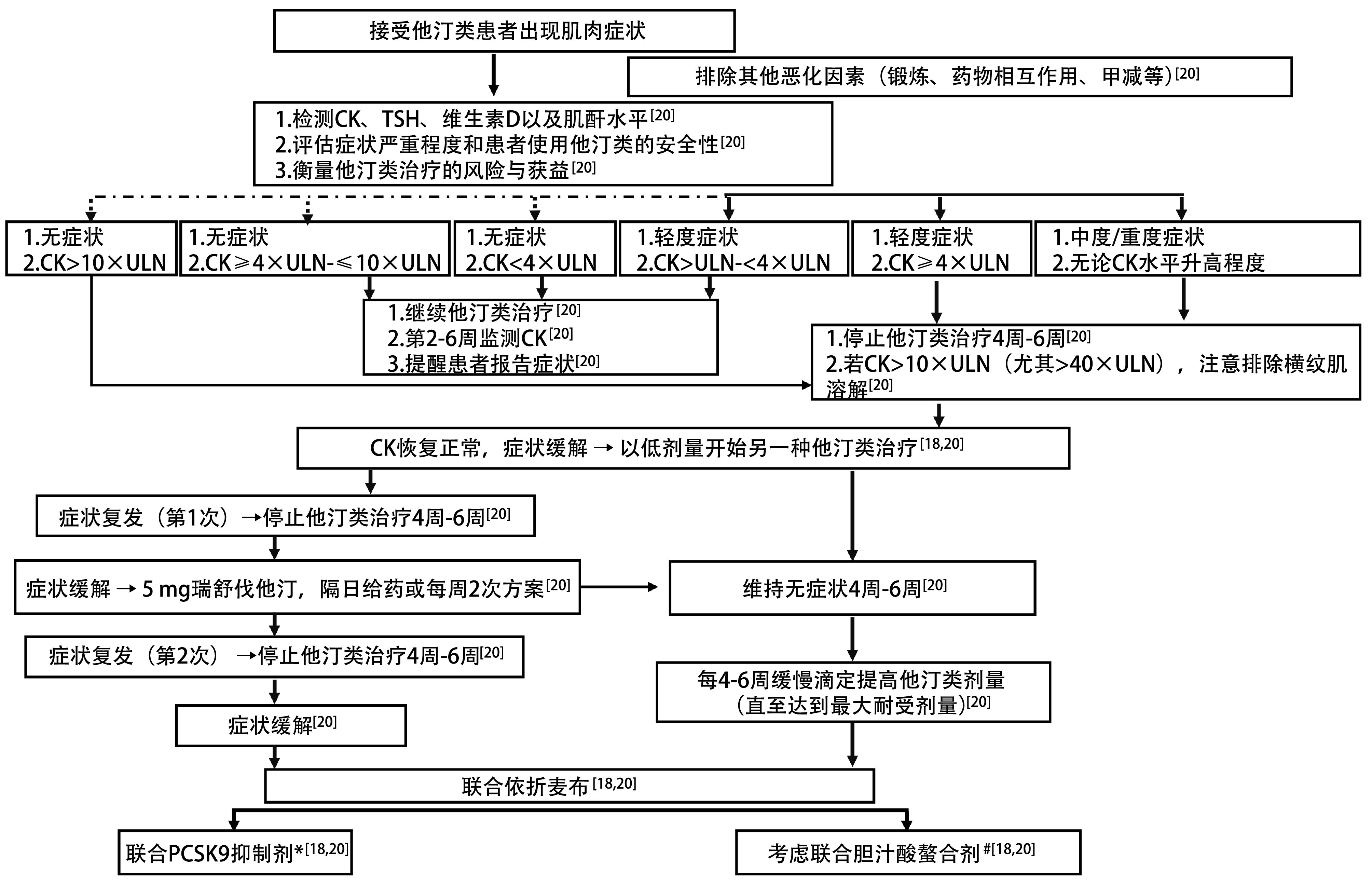
他汀类药物治疗期间肌肉症状的管理流程^[18, 20]^。^*^若患者4周后LDL-C>100 mg/dL且伴心血管疾病，或>130 mg/dL且伴高危因素^[20]^；^#^如，考来维仑、考来替泊或考来烯胺^[18, 20]^。CK：肌酸激酶；LDL-C：低密度脂蛋白胆固醇；PCSK9：前蛋白转化酶蛋白酶/kexin9型；TSH：促甲状腺激素；甲减：甲状腺功能减退。

### 2.2 CNS反应

#### 2.2.1 发生率和临床特点

接受洛拉替尼治疗的患者可能出现多种CNS反应，包括癫痫发作、精神影响以及认知功能（如意识、记忆、时空定位、注意力等）、情绪（包括自杀意念）、言语、精神状态和睡眠改变^[7, 9]^。总体发生率为35%（*n*=149）^[22]^，至首次发生任何CNS反应的中位时间为1.4个月（*n*=476）^[7]^。CNS反应中，认知与情绪影响最为常见，任何级别和3级-4级发生率分别为21%和2%（认知影响）、16%和2%（情绪影响）^[13]^（*n*=149），次之为言语和精神影响^[7]^。认知、情绪、言语和精神影响的至发生中位时间分别为109 d、43 d、49 d和23 d，持续中位时间分别为223 d、143 d、147 d和74 d^[15]^。CNS反应导致的剂量延迟、减量或永久性终止治疗发生率分别为10%、8%和2.1%（*n*=476）^[7]^。

中国患者中的CNS AE发生率（6.4%）低于总人群，认知影响、情绪影响、言语影响发生率分别为2.8%、1.8%、0.9%。大多数CNS AE为治疗相关的，除外言语影响。绝大多数CNS AE为1级或2级，未导致治疗终止^[14]^。

此外，基于临床专家的经验表明，发生CNS反应的患者常表现为言语模式的改变，减缓为正常语速的75%，也有认知的变化以及情绪的改变，抑郁并不多见。当然，患者的家属或照护者更熟悉其情绪或行为模式，他们更容易觉察患者的变化^[23]^。

#### 2.2.2 评估与分级

CNS反应可能源于系统性或针对CNS转移灶的治疗（如手术、放疗、药物等）ADR，也可能由肿瘤本身引起，同时也受到临床、心理社会、遗传因素以及认知储备的影响^[24]^。对比增强磁共振成像（magnetic resonance imaging, MRI）可识别或区分新病灶与经治CNS转移灶、既往放疗引起的脑部异常与非肿瘤相关的脑血管病理改变。患者精神状态评估方法包括患者自评量表，如抑郁量表Symptom Checklist 90（SCL-90）、Beck Depression Inventory（BDI）^[25]^、简易认知量表Mini-Cog^[26]^以及开放式与封闭式问卷^[9]^。语言方面的评估工具通常相对复杂，建议医生注意与患者的沟通，以临床判断为宜。CNS反应分级标准可参照美国国立癌症研究所（National Cancer Institute, NCI）发布的不良事件通用术语标准（Common Terminology Criteria for Adverse Event, CTCAE）5.0版本（NCI CTCAE v5.0）（表 4）^[27]^。表 4列出了认知、情绪、言语和精神效应类别下常见子类的分级标准。

**表 4:** 中枢神经系统病变分级标准

CACTE术语	1级^[27]^	2级^[27]^	3级^[27]^	4级^[27]^	5级^[27]^
认知功能改变					
记忆障碍	轻度记忆障碍	中度记忆障碍；工具性日常生活活动受限	重度记忆障碍；自我照顾日常生活活动受限	-	-
意识模糊	轻度定向障碍	中度定向障碍；工具性日常生活活动受限	重度定向障碍；自我照顾日常生活活动受限	危及生命的后果；有紧急干预指征	-
情绪改变					
焦虑	轻度症状；无干预指征	中度症状；工具性日常生活活动受限	重度症状；自我照顾日常生活活动受限；有住院指征	危及生命的后果；有紧急干预指征	-
易怒	轻度症状；容易安慰	中度；工具性日常生活活动受限；有需要更多关注的指征	重度异常或过度反应；自我照顾日常生活活动受限；无法安慰；有医疗或精神干预指征	-	-
抑郁	轻度抑郁症状	中度抑郁症状；工具性日常生活活动受限	重度抑郁症状；自我照顾日常生活活动受限；无住院指征	危及生命的后果，威胁伤害自己或他人；有住院指征	死亡
语言功能改变					
构音障碍	轻度口齿不清	中度发音障碍或口齿不清	重度发音障碍或口齿不清	-	-
根据NCI CTCAE v5.0^[27]^。后同。					

#### 2.2.3 监测与管理

启动治疗前与患者、家属以及照护者沟通，告知若患者出现思考障碍、情绪变化、幻觉、癫痫、言语或睡眠变化等时需立即报告。治疗前和治疗期间定期通过影像学监测CNS状态（开始治疗后每6周一次直至30个月，之后每3个月一次）。若患者有精神病史，那么需评估其既往治疗方案以及最末次用药与开始洛拉替尼的时间间隔。出现CNS反应时，应根据严重程度调整洛拉替尼剂量^[7]^。大多数CNS AE能够在调整剂量或不进行临床干预的情况下缓解。剂量调整是CNS AE的有效管理方式，且不影响疗效。研究^[22]^显示16周内减量与未减量亚组相比，以及相对剂量强度≥平均值与<平均值亚组相比，12个月PFS率均无显著差异（分别为93% *vs* 89%；90% *vs* 93%）。若CNS反应影响患者日常生活，则有必要进行治疗干预，需根据药物相互作用来选择对洛拉替尼影响最小的精神科药物（图 3）^[9]^。

**图 3:**
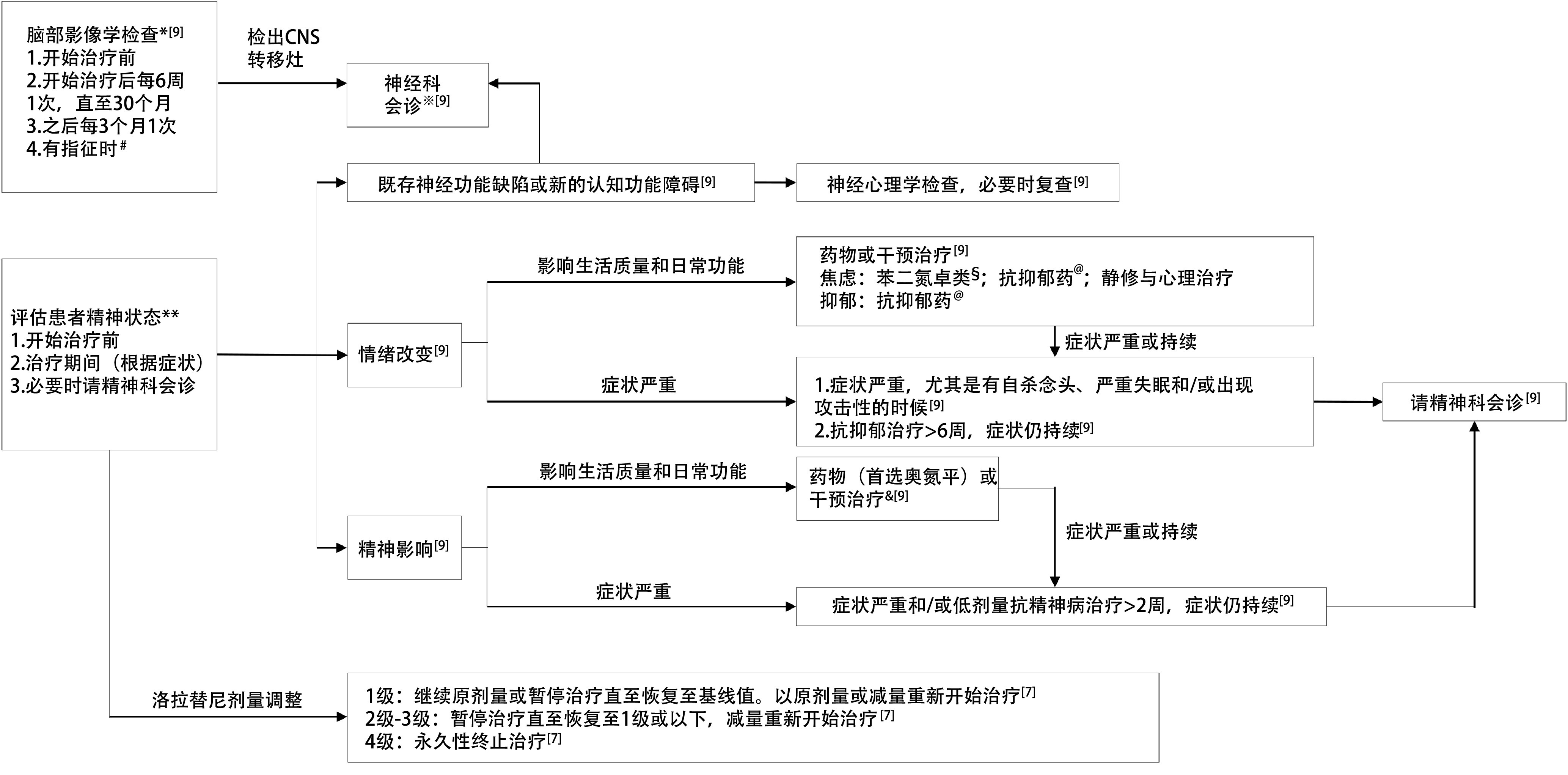
洛拉替尼相关CNS反应的管理流程。^*^首选对比增强MRI，次选CT^[9]^；^#^检出新的神经系统症状，或在CNS以外的部位出现肿瘤进展^[9]^；^※^旨在评估新的或先前CNS转移灶对认知和整体功能的影响，以判断或排除是否与洛拉替尼治疗相关^[9]^；^**^对于既存病变的患者，应在治疗前与本人及其看护者充分沟通，并鼓励其及时报告可能的认知改变或精神症状^[9]^；^§^应避免使用阿普唑仑和咪达唑仑（均经CYP3A4/5代谢）；慎用安定和氟硝西泮；其他药物在这种情况下耐受性良好^[9]^；^@^首选度洛西汀、安非他酮和阿戈美拉汀；其他药物慎用；抗抑郁药通常2周-3周起效，不建议在此期间调整洛拉替尼剂量^[9]^； ^&^ 避免使用喹硫平和齐拉西酮（均经CYP3A4/5代谢）；慎用利培酮和氯氮平；抗精神病药物通常≥1周起效，期间可使用抗焦虑药作为支持治疗，洛拉替尼剂量调整应在此期间过后才考虑^[9]^。CNS：中枢神经系统；MRI：磁共振成像；CT：计算机断层扫描。

### 2.3 周围神经病变

#### 2.3.1 发生率和临床特点

洛拉替尼治疗患者中常见周围神经病变，典型症状为四肢刺痛、麻木和疼痛，至发生的中位时间为7 d^[9, 19]^。中国患者中报告的发生率[任何级别和3级-4级：17%（治疗相关11%）和0%]低于总人群（34%和2%）^[13, 14]^。周围神经病变常与体重增加和/或水肿重叠，11.4%的患者同时报告上述三种AE^[19]^。周围神经病变相关的暂停治疗或减量发生率均为4.1%^[19]^。

#### 2.3.2 评估与分级

周围神经病变适用的评估工具包括毒性反应分级量表[如NCI CTCAE v5.0（表 5）等]、临床神经学检查、评分量表（如神经病变总评分）以及患者报告的结果测评（如调查问卷等）^[28]^。

**表 5:** 周围神经病变分级标准

CACTE术语	1级^[27]^	2级^[27]^	3级^[27]^	4级^[27]^	5级^[27]^
周围运动神经病变或周围感觉神经病变	无症状，仅为临床或诊断学发现	中度症状；工具性日常生活活动受限	重度症状；自我照顾日常生活活动受限	危及生命；需紧急干预	死亡（对于周围运动神经病变）

#### 2.3.3 监测与管理

开始洛拉替尼治疗前，需明确患者是否因其他基础疾病或先前化疗而既存周围神经病变及其相关的功能缺陷^[9]^；治疗期间每6-8周以及后续随访时定期监测^[21]^。对于3级或4级周围神经病变，应参照针对非特异性ADR的剂量调整方案，暂停洛拉替尼直至症状缓解至≤2级或基线值，之后减量继续治疗^[7]^。身体功能缺陷管理包括药物、运动和物理或职业治疗干预、转诊至康复训练服务中心、强化安全监督^[28]^。针对疼痛性周围神经病变的药物首选普瑞巴林和加巴喷丁，逐步滴定至最大耐受剂量。需注意，二者可导致头晕、困倦和步态障碍，镇静安定类或抗抑郁药能够增强这种镇静效果。另外，加巴喷丁与阿片类药物同时使用时，可发生呼吸抑制^[9]^。尚未确立针对非疼痛性病变的有效手段^[28]^。

### 2.4 水肿

水肿为洛拉替尼治疗的患者中最常报告的ADR之一，多为轻度，以外周性水肿占比最大^[19]^。中国患者中发生率[任何级别和3级-4级：28%（治疗相关27%）和0.9%]低于总人群（56%和4%）^[13, 14]^。至首次发生水肿的中位时间为42 d，持续中位时间为163 d^[19]^。分别有5.8%、6.1%的患者因水肿而暂停或减量治疗（*n*=295, NCT01970865）^[19]^。

当出现水肿时，首先应排除其他潜在诱因，如心源性、其他药物、低蛋白血症、甲状腺功能减退、肢体血管栓塞等。建议轻中度患者采取保守的物理措施，如使用压缩绷带或长袜、抬高下肢，以及改善生活方式如限盐、加强体育锻炼等。重度水肿情况下，尤其显著影响生活质量或表现为肺水肿时，可短期使用最小有效剂量的呋塞米。必要时洛拉替尼减量或暂时停药，密切观察^[9, 21, 29]^。

### 2.5 体重增加

#### 2.5.1 发生率和临床特点

体重增加在中国患者中发生率[任何级别和3级-4级：54%（治疗相关32%）和6.4%]总体上高于总人群（38%和17%），但3级-4级相对较少^[13, 14]^。通常报告于洛拉替尼开始治疗后2个月内，至发生中位时间为64 d^[19]^。30.9%的患者增加基线期体重的10%-20%，13.5%的患者增加 >20%。相对于基线期体重最大增幅百分比中位值为11.4%（*n*=295, NCT01970865）^[19]^。

#### 2.5.2 体重增加分级标准

可参照NCI CTCAE v5.0（表 6）^[27]^。

**表 6:** 体重增加分级标准

CACTE术语	1级^[27]^	2级^[27]^	3级^[27]^	4级^[27]^	5级^[27]^
体重增加	与基线值相比增加5%-<10%	与基线值相比增加10%-<20%	与基线值相比增加≥20%	-	-

#### 2.5.3 监测与管理

制定治疗方案之初应充分告知患者他们可能会经历某种程度的体重增加，以确保患者做好准备进行及时的饮食调整和适当的体育锻炼^[19]^。推荐每次随访时监测体重，每2个月咨询营养师，以纠正潜在的依从性问题^[19, 21]^。

### 2.6 肝毒性

#### 2.6.1 发生率和临床特点

丙氨酸转氨酶（alanine aminotransferase, ALT）和/或天冬氨酸转氨酶（aspartate aminotransferase, AST）升高在中国患者中任何级别发生率（超过40%）高于总人群（14%-17%），3级-4级发生率相当（均为2%-3%）^[13, 14]^。

#### 2.6.2 评估与分级

肝毒性分级可参照NCI CTCAE v5.0（表 7）^[27]^。

**表 7:** 肝毒性分级标准

CACTE术语	1级^[27]^	2级^[27]^	3级^[27]^	4级^[27]^	5级^[27]^
ALT升高和AST升高	>ULN-3.0×ULN（基线值正常时）；1.5-3.0×基线值（基线值异常时）	>3.0-5.0×ULN（基线值正常时）； >3.0-5.0×基线值（基线值异常时）	>5.0-20.0×ULN（基线值正常时）； >5.0-20.0×基线值（基线值异常时）	>20.0×ULN（基线值正常时）； >20.0×基线值（基线值异常时）	-

#### 2.6.3 监测与管理

ALK TKI相关的肝毒性常导致减量或治疗暂停，但潜在的影响因素研究十分有限，目前发现的风险因素包括高龄、女性、肝病或肝炎病毒感染、H2拮抗剂或质子泵抑制剂的使用等^[30]^。对于3级-4级肝毒性，暂停治疗通常能够逆转。应参照针对非特异性ADR的剂量调整方案，暂停洛拉替尼直至症状缓解至≤2级或基线值，之后减量继续治疗^[7]^。

### 2.7 高血压

#### 2.7.1 发生率和临床特点

高血压见于18%的患者，3级-4级为10%（分级见表 8）；中国患者中发生率略低（12%）^[13, 14]^。至发生中位时间为208 d（约6.8个月），持续中位时间为219 d（约7.2个月）^[15]^。2.3%的患者因高血压而停用洛拉替尼（*n*=476）^[7]^。

**表 8:** 高血压分级标准

CACTE术语	1级^[27]^	2级^[27]^	3级^[27]^	4级^[27]^	5级^[27]^
高血压（成人）	收缩压120 mmHg-139 mmHg，或舒张压80 mmHg-89 mmHg	若既往在正常范围内，收缩压140 mmHg-159 mmHg，或舒张压90 mmHg-99 mmHg；相对于基线值的变化需要医疗干预；复发或持续（≥24 h）；症状性血压升高 >20 mmHg（舒张压）或达到 >140 mmHg/90 mmHg；有单药治疗指征	收缩压≥160 mmHg，或舒张压≥100 mmHg；需要医疗干预；有使用多种药物或比既往方案更强化的治疗指征	危及生命的后果（如恶性高血压、暂时性或永久性神经功能缺陷、高血压危象）；有紧急干预指征	死亡

#### 2.7.2 评估与分级

高血压分级可参照NCI CTCAE v5.0（表 8）^[27]^。

#### 2.7.3 监测与管理

开始洛拉替尼治疗前应控制血压，将血压控制到<130 mmHg/80 mmHg。可起始采取联合降压药物（单片固定复方制剂）治疗，并在治疗后2周时以及之后至少每个月监测诊室血压^[7]^，鼓励患者家庭自测血压监测，必要时24 h动态血压监测。给予降压治疗时，需注意动脉粥样硬化、动脉瘤、心力衰竭、代谢综合征、慢性肾衰竭和视网膜病变等并发症^[31]^。若经最佳医疗措施仍不能充分控制，需永久性终止洛拉替尼治疗^[7]^。

### 2.8 高血糖

#### 2.8.1 发生率和临床特点

高血糖在中国患者中发生率[任何级别和3级-4级：18%（治疗相关17%）和2.8%]高于总人群（9%和3.2%；*n*=476）^[7, 14]^。至发生中位时间为145 d，持续中位时间为113 d^[15]^。0.8%的患者因高血糖而永久性终止洛拉替尼治疗（*n*=476）^[7]^。

#### 2.8.2 评估与分级

高血糖分级可参照NCI CTCAE v5.0（表 9）^[27]^。

**表 9:** 高血糖分级标准

CACTE术语	1级^[27, 33]^	2级^[27, 33]^	3级^[27, 33]^	4级^[27, 33]^	5级^[27, 33]^
高血糖	异常血糖高于基线值，无药物干预指征（空腹血糖 >ULN-160 mg/dL或 >ULN-8.9 mmol/L^*^）	与基线期相比加强对糖尿病的日常管理；有开始口服降糖药的指征；进行糖尿病检查（空腹血糖 >160 mg/dL-250 mg/dL或 >8.9 mg/dL-13.9 mmol/L^*^）	有使用胰岛素指征；有住院指征（空腹血糖 >250 mg/dL-500 mg/dL或 >13.9 mg/dL-27.8 mmol/L^*^）	危及生命的后果；有紧急干预指征（空腹血糖 >500 mg/dL或 >27.8 mmol/L^*^）	死亡
^*^血糖具体数值参考CTCAE v4.03^[33]^。					

#### 2.8.3 监测与管理

开始治疗前应测量空腹血糖，治疗期间需定期监测。出现高血糖时，需及时给予降糖治疗，以避免视网膜病、肾病和心血管疾病等并发症^[32]^。若经最佳医疗措施仍不能充分控制，需永久性终止洛拉替尼治疗^[7]^。

### 2.9 房室传导阻滞

#### 2.9.1 发生率和临床特点

房室传导阻滞是从心房到心室的冲动传递延迟或中断^[34]^。接受洛拉替尼治疗的患者，1.9%会发生房室传导阻滞，0.2%为3级并置入了起搏器（*n*=476）^[7]^。

#### 2.9.2 评估与分级

房室传导阻滞可以是部分或完全的：一度和二度为部分阻滞，三度为完全阻滞^[33]^。各个度有独立的分级，可参照NCI CTCAE v5.0（表 10）^[27]^。

**表 10:** 房室传导阻滞分级标准

CACTE术语	1级^[27]^	2级^[27]^	3级^[27]^	4级^[27]^	5级^[27]^
一度房室传导阻滞	无症状，无干预指征	无紧急干预指征	-	-	-
二度房室传导阻滞	无症状，无干预指征	有症状；有药物干预指征	有症状且药物无法完全控制，或医疗设备（如起搏器）可以完全控制；新发（仅适用于Mobitz II型）	危及生命；有紧急干预指征	死亡
三度（完全）房室传导阻滞	-	无紧急干预指征	有症状且药物无法完全控制，或医疗设备（如起搏器）可以完全控制；新发	危及生命；有紧急干预指征	死亡
洛拉替尼剂量调整取决于房室传导阻滞的程度（部分或完全），而非分级。									

#### 2.9.3 监测与管理

开始洛拉替尼治疗前、治疗期间每4个月（伴临床显著心脏事件易感因素的患者每个月）以及有低灌注警示症状时（如头晕或晕厥、心悸或胸痛、应用新的或改变了原有的心血管药物）应监测心电图及24 h动态心电图监测^[7, 9, 19, 34]^。一旦检出房室传导阻滞和/或结构性心脏病变，出现二度或三度房室传导阻滞的患者应转诊至心血管科以行更明确的检查^[9, 34]^。一度和无症状二度（Mobitz I型）患者可继续日常活动但避免导致PR间期延长的药物，二度（Mobitz II型）且症状持续和三度患者有必要安装起搏器^[15, 34]^。若出现三度复发，也可考虑永久性终止治疗^[7]^。需警惕起搏器感染常见于老年人，特别是有基础疾病者^[34]^。

### 2.10 间质性肺疾病（interstitial lung disease, ILD）或非感染性肺炎

#### 2.10.1 发生率和临床特点

ALK TKI在NSCLC患者中诱导的ILD或非感染性肺炎常见的模式为隐源性机化性肺炎，其次是急性间质性肺炎和过敏性肺炎^[35]^。尽管罕见，ILD或非感染性肺炎可导致致命性并发症^[36]^。洛拉替尼治疗的患者中任何级别和3级-4级ILD或非感染性肺炎发生率分别为1.9%、0.6%，0.8%的患者因ILD或非感染性肺炎终止治疗（*n*=476）^[7]^。目前缺乏在中国人群中的数据。ILD的风险因素可能包括吸烟史、既往ILD以及胸腔积液^[36]^。

#### 2.10.2 评估与分级

若患者出现提示ILD或非感染性肺炎的征象，如呼吸困难、咳嗽和发热等症状恶化，应立即进行诊断性检查^[7]^。计算机断层扫描（computed tomography, CT）能够区分ILD为慢性、亚急性或急性^[37]^。ILD或非感染性肺炎的分级标准可参照NCI CTCAE v5.0（表 11）^[27]^。

**表 11:** 间质性肺疾病或非感染性肺炎分级标准

CACTE术语	1级^[27]^	2级^[27]^	3级^[27]^	4级^[27]^	5级^[27]^
非感染性肺炎（累及肺实质的局灶性或弥漫性炎症）	无症状，仅为临床或诊断学发现，无需干预	有症状，需要医疗干预；工具性日常生活活动受限	重度症状；自我照顾日常生活活动受限，有吸氧指征	危及生命的呼吸功能损伤，需紧急干预（如气管切开或插管）	死亡

#### 2.10.3 监测与管理

疑诊ILD或非感染性肺炎时，应立即暂停洛拉替尼治疗。一旦确诊，应永久性终止治疗^[7]^。药物诱导肺病变的治疗选择十分有限，需肺科、心血管科、放射科、病理科以及风湿科等多学科团队合作。即使停药，慢性纤维化病变基本上是不可逆的^[38]^。对于≥2级ILD或非感染性肺炎，应给予皮质类固醇并考虑经验性抗生素或支气管肺泡灌洗；3级-4级应住院治疗，在上述措施基础上进行支气管镜检和/或活检、使用免疫抑制剂、给予吸氧和重症监护等^[37]^。

## 3 药物相互作用

洛拉替尼经肝代谢，主要参与酶为细胞色素P450（CYP）3A4和尿苷二磷酸葡萄糖醛基转移酶（UGT）1A4代谢。另外，洛拉替尼自身为CYP3A和P-糖蛋白的中效诱导剂。当患者在相同时期接受针对基础疾病或ADR的治疗干预时，必须注意药物相互作用，禁忌与CYP3A强效诱导剂同时给药（表 12）^[7, 15]^。

**表 12:** 禁忌或避免与洛拉替尼同时使用的药物

药物类别	相互作用^[7, 15]^	注意事项^[7, 15]^	备注^[7, 15]^
CYP3A强效诱导剂	使洛拉替尼血浆浓度↓、有效性↓	禁忌	开始洛拉替尼治疗之前，任何CYP3A强效诱导剂需停用该诱导剂的3个血浆半衰期；与利福平同时使用可导致健康受试者发生重度肝毒性反应
CYP3A中效诱导剂	使洛拉替尼血浆浓度↓、有效性↓	避免； 若必须同时给药，洛拉替尼剂量应增至125 mg	-
CYP3A强效抑制剂	使洛拉替尼血浆浓度↑、ADR发生率和严重程度↑	避免； 若必须同时给药，洛拉替尼剂量应减至75 mg	若因患者ADR已将洛拉替尼剂量减至75 mg，应进一步降低至50 mg；若患者停用CYP3A强效抑制剂，在该抑制剂的3个血浆半衰期后，将洛拉替尼升至开始CYP3A强效抑制剂治疗之前的剂量水平
CYP3A中效抑制剂	使洛拉替尼血浆浓度↑、ADR发生率和严重程度↑	避免； 若必须同时给药，洛拉替尼剂量应减至75 mg	代表药物为氟康唑，该药也是CYP2C9中效抑制剂、CYP2C19同工酶的强效抑制剂
治疗窗窄的CYP3A底物	使CYP3A底物血浆浓度↓、有效性↓	避免； 若必须同时给药，根据CYP3A底物批准的成分标签增加剂量	-
治疗窗窄的P-gp底物	使P-gp底物血浆浓度↓、有效性↓	避免； 若必须同时给药，根据P-gp底物批准的成分标签增加剂量	-
洛拉替尼自身为CYP3A4/5和P-gp的中效诱导剂。P-gp：P糖蛋白。			

## 4 总结

洛拉替尼为*ALK*阳性晚期NSCLC患者提供了新颖且高效的治疗选择，无论作为一线方案，还是一代或二代ALK TKI经治患者的后线用药，均获得了令人瞩目的疗效，而且对CNS病灶也有强大的抗肿瘤活性。洛拉替尼总体上具有良好的安全性，但其毒性反应特征与既往ALK TKI不同。最常见的ADR或AE为高脂血症与CNS反应，多为轻至中度，通常经剂量调整和/或标准医疗处理即可管理，很少导致永久性停药。鉴于洛拉替尼的ADR或AE涉及不同器官系统，多学科团队合作是至关重要的。对于*ALK*阳性晚期NSCLC，开始治疗前应充分评估患者基线特征与用药状况，预先告知可能发生的毒性反应，并持续监测获益-风险平衡，以达到及时发现和有效管理。
